# Reply to: Genome-wide association studies of polygenic risk score-derived phenotypes may lead to inflated false positive rates

**DOI:** 10.1038/s41598-023-29953-7

**Published:** 2023-03-14

**Authors:** Rita Guerreiro, Jose Bras

**Affiliations:** Department of Neurodegenerative Science, Van Andel Institute, Grand Rapids, MI USA

**Keywords:** Genome-wide association studies, Genetic markers

**replying to**: E. Uffelmann; *Scientific Reports* 10.1038/s41598-023-29428-9 (2023).

## Introduction

In their Matters Arising article, Uffelmann et al.^1^ present simulation experiments suggesting that the approach used in^2^, where genome-wide association studies are based on polygenic risk score-derived phenotypes, may lead to inflated positive rates. We acknowledge the importance of confirming the increased number of false positive results by performing simulation experiments but have reservations about how these impact our interpretation of data and subsequent conclusions derived from the results in the original study.

Our study determined each individual's polygenic risk score (PRS) for AD in the UK Biobank dataset. Using individuals within the extreme risk distribution, we performed a GWAS based solely on known genetic risk for disease, independently of diagnoses. Given that we were separating the two study groups by their genetics and not a well-defined trait or phenotype, we observed an excess of genome-wide significant associations (p < 5 × 10^–08^), shown by a genomic inflation factor of 2.442. Since we expected this to occur (given the study design), we did not focus on these variants in our discussion and do not claim that the 246 independent loci are significantly associated with AD. Consequently, we do not consider this an increase in the number of loci associated with the disease. Instead, we adopted a more stringent threshold (p < 1 × 10^–15^) to highlight highly significant variants associated with the published PRS extremes and potentially avoid false positives that were expected due to, for example, LD with our initial risk variants in the PRS calculation.

Our view is that we should not use these results as those from a typical GWAS since p-values will generally be inflated/deflated for variants that were/were not associated in the base GWAS. Additionally, the effects will not translate to the typical case/control paradigm since variant frequencies will not necessarily reflect those in the general (control) population. We carefully considered these caveats before performing the study, and the results matched our expectations.

A critical aspect of this work is that the study design we adopted is a step forward in allowing us the possibility of auditing previously established associations. For example, despite using summary statistics from^3^, several loci reported as genome-wide significant in that manuscript are not significant in our study. Examples of these are variants in loci nominated as *HESX1, HS3ST1, BZRAP1-AS1, SUZ12P1, and ALPK2*. Interestingly, in a follow-up publication to the original GWAS^4^, the same group did not find associations at these signals either, after increasing their case sample size by 25% and their controls by approximately threefold. This was true despite using the same base data published in the initial GWAS. Had we removed these variants from our analysis, as Uffelmann et al. suggest, we would not have been able to determine that they were false positives in the base GWAS and, more generally, would have missed the possibility of auditing the originally published loci.

Additionally, new loci recently identified for AD are known to be involved in other related diseases. Examples are *TMEM106B* or *GRN*. Two cases can be made for this type of finding: first, that the associations reflect true pleiotropic events that underlie clinically and pathologically distinct diseases; or second, that by dramatically increasing the sample size, one is inadvertently including a larger number of misdiagnosed cases, leading to variants from a different disease to be identified as associated with the disease of interest. The latter point has been well illustrated by others^5,6^. As long as this is not an issue in the base GWAS used to calculate the PRS, the proposed study design will not suffer from this drawback and will instead allow us to identify such associations.

Finally, among the loci we highlight as significant are genes not previously associated with AD by GWAS, even in the base data used to generate the PRS, but that were associated with the disease through other study designs. Examples of these are *IL34, ACE,* and *KANSL1*. Despite not being significant in the base GWAS used in our study, these loci reached p-values lower than 1 × 10^–15^ in the PRS extreme approach. They have also been associated with AD in larger, more recent, independent studies, showing the validity of this approach in these cases.

Our original publication does not claim to improve the power for a GWAS of Alzheimer’s disease. Instead, we are performing an association study agnostic to phenotype to determine if that can help obtain new variants associated with a genetic risk profile for AD. There are currently several false positive loci in the typical GWAS for AD, which is a problem that needs to be addressed, and we propose that a study in PRS extremes may be one way to start addressing that. As with any approach proposed initially, we fully recognize shortcomings and aspects that need to be refined to help clarify the methodology and results, and so we welcome the work of Uffelmann et al. We would also welcome developing specific statistical approaches that distinguish false and true positives from such analyses.

In conclusion, we appreciate the authors' effort to use simulations to demonstrate the presence of false positives in these results, which could be inferred from the metrics we presented in our study. We agree that phenotype definitions based on PRSs require careful consideration in any GWAS aimed at identifying novel significant loci associated with disease, which was not the main goal of our original study. Importantly, the interpretation of results and the analyses performed in our manuscript have considered the presence of false positives. Even though (or maybe due to) not fitting the typical statistical paradigms in the field, the results and interpretations presented show us the need for systematic auditing of currently associated loci in AD and the need to develop analytical methodologies to do this when typical replication studies are not feasible. We welcome the work of Uffelmann et al. and hope this drives further interest in this area.
